# Meristem fate: to terminate, or not?

**DOI:** 10.3389/fpls.2025.1739587

**Published:** 2026-01-19

**Authors:** Chan-Yi Ivy Lin, Vivian F. Irish

**Affiliations:** 1 Department of Molecular, Cellular and Developmental Biology, Yale University, New Haven, CT, United States; 2 Department of Ecology and Evolutionary Biology, Yale University, New Haven, CT, United States

**Keywords:** determinacy, development, indeterminacy, meristem, WUSCHEL

## Abstract

Plants exhibit remarkable physiological and developmental flexibility, enabling continued organogenesis, adaptation to environmental stimuli, and underpinning a vast diversity of shoot architectures. Central to this capacity is the plasticity of plant meristems, that function as dynamic sources of stem cells and define pivotal decision points between indeterminate (sustained) and determinate (finite) growth. Understanding the regulatory networks governing meristem fate is critical for both basic plant science and practical applications in crop architecture and yield optimization. In this review, we focus on the interplay between transcriptional regulators and phytohormone gradients that govern the switch between indeterminacy and determinacy in developing shoot meristems. We explore how regulatory networks converge to produce determinate structures such as flowers and thorns in different angiosperm species.

## Introduction

Plant growth is sustained by stem cell populations located in the shoot apical meristem (SAM) and the root apical meristem (RAM), which possess an intrinsic capacity for continuous organogenesis. Key transcription factors, including *WUSCHEL* (*WUS*) in the SAM and *WUSCHEL-RELATED HOMEOBOX5* (*WOX5*) in the RAM (Tvorogova et al., 2021; Shpak and Uzair, 2025), maintain stem cell identity by promoting proliferation and preventing premature differentiation. This proliferative activity underpins the indeterminate nature of the SAM and RAM, enabling persistent growth and developmental plasticity. In this review we focus on shoots; the balance between determinacy and indeterminacy in roots has been covered in several excellent overviews (Shishkova et al., 2008; Lee et al., 2013).

Within the shoot system, the SAM can generate both indeterminate and determinate structures, which diverge in their proliferative capacity and developmental trajectories (Steeves and Sussex, 1989). Indeterminate meristems, such as the primary shoot apical meristem or the axillary meristems, retain the ability to initiate new organs and sustain shoot growth throughout the lifespan of a plant. In contrast, determinate meristems are defined by their commitment to terminal differentiation with limited or finite proliferative capacity. Once specified, determinate meristems undergo limited additional cell divisions and form structures of defined size and organization. In response to floral inductive cues, shoot meristems can convert to determinate floral meristems (FMs) and produce the floral organs prior to termination, while during vegetative growth, axillary meristems can undergo a transition to determinacy, resulting in the formation of thorns.

The distinction between indeterminate and determinate growth is fundamental to plant form and adaptability. Indeterminate meristems confer developmental flexibility, allowing plants to continuously produce new organs and adjust their architecture in response to environmental and hormonal cues. This open-ended growth pattern enables traits such as branching, climbing, and perenniality (Cutri et al., 2013; Maurya et al., 2020; Yang et al., 2023). By contrast, determinate meristems represent developmental endpoints; once a flower or thorn is specified, its proliferative capacity will eventually be terminated, ensuring the formation of specialized structures (Sablowski, 2007; Alvarez et al., 2016; Zhang et al., 2020). The shift from indeterminate to determinate growth thus represents a key developmental decision that balances plasticity and specialization.

## Meristem organization and the basis of indeterminacy

**Figure 1:**
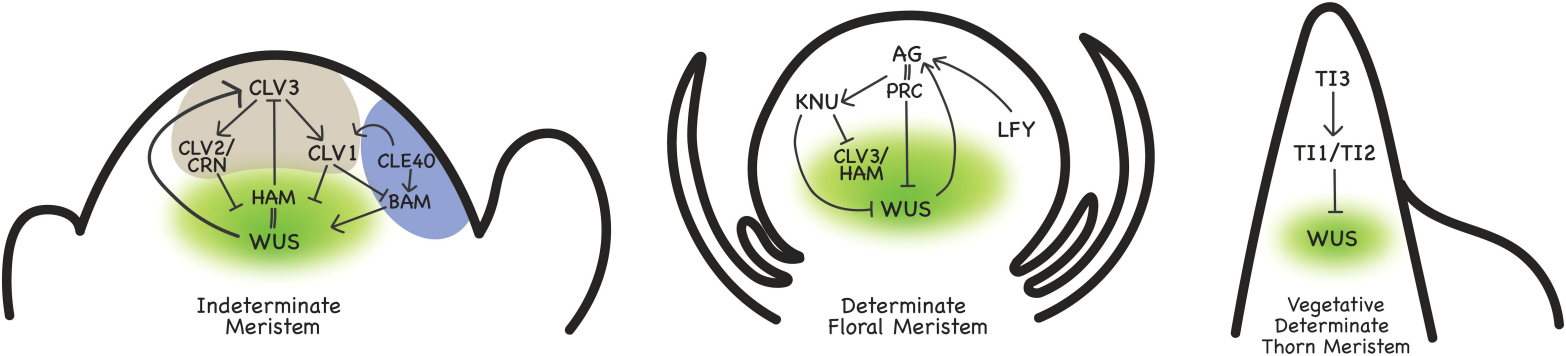
Regulation of indeterminate and determinate meristems. Genes with characterized genetic and/or biochemical interactions are shown. WUS is expressed in the organizing center (OC, green), peripheral zone (PZ) indicated in blue, central zone (CZ) indicated in grey. Arrows depict positive regulation, and bars depict negative regulation. Double lines represent interactions.

In angiosperms, the SAM generally consists of a few hundred cells that are organized into a dome consisting of three clonally distinct cell layers (Steeves and Sussex, 1989). Within this population, different zones of activity can be defined: the organizing center (OC) consisting of only a few cells overlaid by the central zone (CZ), containing a pool of slowly dividing stem cells, while cells in the peripheral zone (PZ) give rise to the lateral organs (**Figure 1**).

The maintenance of indeterminate growth relies on the negative feedback loop between *WUS* and *CLAVATA3* (*CLV3*) in the SAM (Agarwal et al., 2022). *WUS* is expressed in the OC and encodes a homeodomain transcription factor that is required to maintain stem cell activity in the SAM (Jha et al., 2020). WUS is bifunctional and can act as either an activator or a repressor (Ikeda et al., 2009; Yadav et al., 2013). WUS acts as a transcriptional repressor through the interaction with *TOPLESS* (*TPL*) and *TPL*-related transcriptional co-repressors and can directly repress the expression of differentiation-associated transcription factors (Dolzblasz et al., 2016; Jha et al., 2020).

By contrast, WUS protein maintains stem cell homeostasis through moving via plasmodesmata into the overlying CZ cells and promoting the expression of *CLV3* (Yadav et al., 2011; Daum et al., 2014). In turn, *CLV3* which encodes a ligand, binds to receptors such as CLV1, CLV2 and CORYNE (CRN) in the CZ, restricting *WUS* expression to those cells through a negative feedback loop (Zhu et al., 2010; Shinohara and Matsubayashi, 2015; Plong et al., 2021). A number of other factors also modulate this patterning process, including the HAIRY MERISTEM (HAM) proteins, whose expression serves to restrict *WUS* expression to the OC (Zhou et al., 2018). Furthermore, the balance between the CLV3 signaling pathway emanating from the CZ, and the BARELY ANY MERISTEM (BAM) and CLE40 proteins acting in the PZ, together appear to act to maintain the homeostasis of *WUS* expression in the OC (Schlegel et al., 2021). The interplay of these factors to modulate and maintain WUS activity specifically in the OC is critical for the continued indeterminacy of the SAM (**Figure 1**).

## Diversity of floral determinacy patterns across plant species

The regulatory principles governing the fate of meristems appear to be conserved across flowering plants, yet there is considerable variation in how determinacy is deployed to shape architecture. This is particularly evident in the many variations in when and where determinate FMs are formed.

In *Arabidopsis thaliana*, the SAM remains indeterminate during the vegetative stage, continuously producing leaves and axillary meristems. Upon the floral transition, the SAM converts into an inflorescence meristem (IM), which continues to grow indefinitely while positional cues on its flanks specify primordia to become determinate FMs. This developmental strategy results in a monopodial architecture, in which the main shoot axis elongates and the flowers are produced laterally.

In contrast to a monopodial growth strategy, many species exhibit sympodial growth, in which the modulation of meristem determinacy has been extensively utilized for agricultural optimization. For instance, in tomato (*Solanum lycopersicum*), the SAM becomes determinate, while the adjacent axillary meristem continues to grow in an indeterminate manner for several nodes, and then reiterates this process, producing an alternating cycle of determinate and indeterminate growth. However, mutations in *SELF PRUNING* (*SP*) confer a determinate growth habit, in which the main shoot apex terminates in a flower cluster, have been selected for during domestication because the resulting synchronous fruit maturation facilitates mechanized harvesting (Pnueli et al., 1998). A comparable pattern is observed in cucumber (*Cucumis sativus* L.), where vining cultivars maintain monopodial indeterminate growth and have fruits mature one at a time, while bush-type varieties with sympodial growth produce determinate floral meristems and have been bred to promote uniform fruit production in space-limited settings (Liu et al., 2021). Cereal grasses such as maize (*Zea mays*), rice (*Oryza sativa*) and barley (*Hordeum vulgare*) display a somewhat different pathway to floral determinacy. When grasses transition to flowering, they produce an IM that contains spikelet meristems that in turn produce the FMs. The spikelet meristems can be determinate, producing a fixed number of FMs as in maize or rice, or indeterminate, producing multiple FMs, as in wheat or barley. The determinacy of spikelet meristems, along with the determinacy of FMs, is of considerable interest in that modulation of the fate of these meristems can affect yield (Bommert and Whipple, 2018).

While for many flowering plants the transition to determinacy seems irrevocable, it does not have to be fixed. For *Impatiens*, a short-day species, plants grown under short-day conditions can reverse the formation of a terminal flower by moving back to long-day conditions, demonstrating that determinacy can be plastic and respond to environmental cues rather than being an irreversible developmental switch (Tooke et al., 2005). Furthermore, in some perennial species, meristems can transition between indeterminate vegetative and a determinate reproductive status in a seasonal manner, indicating that meristem identity is malleable (Wang et al., 2009; Albani and Coupland, 2010).

## The molecular basis of floral determinacy

The switch from an indeterminate to a determinate floral state is a critical developmental shift, requiring both the downregulation of genes involved in indeterminacy and the activation of functions required for determinacy and floral differentiation. There appear to be many parallels in the gene regulatory networks regulating these floral determinacy processes across species. In *Arabidopsis*, the mobile florigen signal protein, FLOWERING LOCUS T (FT), is produced in leaves and travels to the PZ in the SAM via the phloem (Corbesier et al., 2007). Once it arrives, it establishes a molecular competition between the *TERMINAL FLOWER*1 (*TFL1*) gene product and the floral transcriptional activators LEAFY (LFY) and APETALA1 (AP1) (Goretti et al., 2020; Zhu et al., 2020; Liu et al., 2021).

The Arabidopsis *TFL1* gene is expressed in the CZ where it has two related roles: regulating inflorescence architecture as well as maintaining the indeterminate state (Bradley et al., 1997).The *TFL1* product, a phosphatidylethanolamine-binding protein (PEBP), acts as a cofactor of the bZIP transcription factor FLOWERING LOCUS D (FD), and this complex represses the transcription of key floral identity genes (Hanano and Goto, 2011). When FT, also encoding a PEPB protein, arrives at the apex, it forms the Florigen Activation Complex (FAC) by binding to FD and competing with TFL1 for this essential partner (Zhu et al., 2020). This FT-FD complex directly binds to the promoter of the floral identity gene *AP1* and activates its expression, tipping the balance away from TFL1-mediated floral repression.

This tip towards determinacy is strengthened by the concurrent activity of *LFY*, whose expression is repressed by TFL1, but activated in response to the FAC which displaces TFL1 from the *LFY* promoter (Zhu et al., 2020). LFY, in turn, acts to modulate chromatin status at the *AP1* locus, by recruiting chromatin remodeling factors and promoting the transcription of *AP1*, thus solidifying the floral determinacy pathway (Jin et al., 2021).

*LFY* also has a fundamental role in downregulating indeterminate development. LFY and WUS both bind to the promoter of *AGAMOUS* (*AG*), a gene required for floral determinacy, coordinately upregulating its expression (Lohmann et al., 2001). In turn, AG negatively regulates the expression of *WUS*, culminating in the downregulation of this key factor required for maintaining the indeterminate state of the meristem. AG also activates the expression of a C2H2 zinc finger repressor, KNUCKLES (KNU), which in turn acts to repress *WUS*, in a negative feedback loop (Sun et al., 2009; Sun et al., 2019). Furthermore, KNU negatively regulates components of the *CLV3* signaling pathway, and disrupts HAM activity (Shang et al., 2021). Additionally, AG acts by recruiting Polycomb Group proteins that confer repressive histone methylation to the *WUS* locus to repress *WUS* expression (Liu et al., 2011). Together, these multiple routes to downregulate *WUS* activity provide a very robust means to support the switch from indeterminate to determinate growth (**Figure 1**).

While homologs of these genes have been found in many other angiosperm species, differences in their tissue- and temporal expression can modulate their roles in regulating the switch to determinacy. For instance, in wild-type tomato, the *TFL1* homolog, *SELF-PRUNING* (*SP*), maintains indeterminacy in the sympodial meristems to allow 3–4 leaves to grow before forming a terminal inflorescence, resulting in continuous, sprawling growth (Pnueli et al., 1998; Kang et al., 2022). *SP* is expressed in a somewhat different pattern as compared to Arabidopsis, which may reflect its distinct roles in sympodial growth (Pnueli et al., 2001). Similarly, in cucumber, the *CsTFL1* gene product regulates flowering and inflorescence architecture in the main branch. CsTFL1 interacts with the product of the miRNA biogenesis gene *Negative on TATA less2* (*CsNOT2a*) to bind to CsFD, and CsFT competes with this binding to initiate determinate growth (Zhao et al., 2018; Wen et al., 2019).

While the grasses possess functional homologs of many of the determinacy genes described above, they have also evolved what appear to be distinct grass-specific pathways that regulate determinacy (Whipple, 2017; Bommert and Whipple, 2018; Yuan et al., 2020; Wang et al., 2022). The *RAMOSA* pathway is required for the specification of spikelet pair meristem determinacy, ensuring they develop into the short, determinate branches of the ear and tassel instead of proliferating into long, indeterminate branches. This pathway includes *RAMOSA1* (*RA1*; encoding a C2H2 zinc finger transcription factor), *RA2* (an LBD transcription factor), and *RA3* (a trehalose-6-phosphate phosphatase) (McSteen, 2006; Gallavotti et al., 2010). RA3 regulates the expression of RA1 and RA2, but this function does not depend on its enzymatic activity, suggesting that RA3 has evolved a new transcriptional regulatory role in the control of meristem determinacy (Claeys et al., 2019).

Due to the relative ease of dissecting meristems in maize and rice, several recent single cell RNA-sequencing analyses have identified genes and regulatory networks that could play pivotal roles in establishing spikelet or floret meristem determinacy. These studies also indicate that epigenetic processes and hormone action may play a more prominent role in determining meristem identity than previously recognized (Zong et al., 2022; Sun et al., 2024; Xu et al., 2025).

## Hormonal regulation of floral determinacy

Phytohormones modulate meristem activity and determinacy both as long-range mobile signals and as local regulators of gene expression and growth, integrating with transcriptional networks to fine tune stem-cell maintenance and organogenesis. The fundamental roles of auxin and cytokinin in regulating meristem activity has been comprehensively reviewed (Lee et al., 2019; Wybouw and De Rybel, 2019; Hong and Fletcher, 2023), and we highlight recent advances that refine our understanding of hormonal control, in particular, auxin, over determinacy decisions. An emerging theme from recent work is that hormones establish cellular competence and growth capacity, while transcription factor-based pathways specify cell fate. In indeterminate meristems, high cytokinin in the CZ and balanced auxin distribution together maintain proliferation and prevent premature differentiation. In the transition to determinate floral meristems, the activation of genes such as *AG, LFY* and *AP1 c*an in turn modulate hormone-related gene expression.

Auxin serves as a central integrator that balances stem cell proliferation in the CZ with organ initiation in the PZ. Moreover, auxin does not simply promote one fate over the other but rather establishes positional information that enables cells to interpret and respond appropriately (Smith and Nimchuk, 2023). For instance, auxin levels can be modulated by CRABS CLAW (CRC), a direct target of *AG*, at auxin biosynthesis loci such as *YUCCA4* (*YUC4*), enabling the increase of auxin production that ultimately drives floral differentiation (Yamaguchi et al., 2017; Yamaguchi et al., 2018).

Once the initiation of lateral organs is established, the auxin peak then acts as an instructive signal to promote cell wall extensibility and activate transcription factors like MONOPTEROS (MP) to execute organ-building programs (Yamaguchi et al., 2013). The repression of *WUS* is also integrated with hormonal signals to secure floral meristem termination. KNU directly represses both the auxin transporter gene *PIN1* and the cytokinin biosynthesis gene *IPT7* by mediating H3K27me3 deposition on these loci to maintain this developmental decision (Silva et al., 2018; Wang et al., 2024). In addition, *AUXIN RESPONSE FACTOR3* (*ARF3*) is expressed in the periphery of the SAM and subsequently can translocate to the OC and repress cytokinin activity and *WUS* expression (Zhang et al., 2022).

## The molecular basis of vegetative determinacy

Less is known about the molecular mechanisms controlling the formation of determinate vegetative meristems, or thorns. In Carrizo citrange (a citrus hybrid), each node bears an indeterminate axillary meristem and an adjacent determinate thorn meristem (Ikeda et al., 2009; Lee et al., 2013; Zhu et al., 2020). This spatial arrangement highlights the remarkable degree of developmental plasticity, where indeterminate and determinate structures arise from closely associated regions, and meristem fate is governed by a complex interplay of positional and molecular cues.

Two TCP transcription factors, THORN IDENTITY1 (TI1) and TI2, are expressed in thorn primordia and are required for thorn development (Zhang et al., 2020). TI1 and TI2 induce the determinate state via downregulating *WUS* expression in citrus thorn meristems, with TI1 having been shown to directly bind to the *WUS* promoter to mediate this repression (Zhang et al., 2020). The citrus *WUS* promoter has a TCP consensus binding site that is not present in *Arabidopsis thaliana*, and mutation of this cis-element disrupts TI1-mediated *WUS* regulation, suggesting that the molecular basis for vegetative indeterminacy, at least in this example, may have been a relatively recent evolutionary innovation (Zhang et al., 2020). *TI1* and *TI2* are transcriptionally upregulated in thorn primordia by TI3, which is a member of the SHI/STY family of transcription factors (Zheng et al., 2026). TI3 may serve a dual function of coordinating the upregulation of the pathway controlling *WUS* downregulation with concomitant regulation of auxin flux, as members of the SHI/STY family have also been shown to promote the expression of several *YUCCA* auxin biosynthesis genes (Eklund et al., 2010).

Recent findings have shown that citrus homolog of *TFL1*, termed *CsCEN*, functions similarly to Arabidopsis *TFL* in ectopic expression studies (Pillitteri et al., 2004; Zhang et al., 2009). Furthermore, *CsCEN*, appears to have a conserved molecular function in maintaining indeterminacy in axillary meristems by repressing *TI1* and *TI2* via interacting with *FD* (Zhang et al., 2021). In addition, overexpression of LFY and AP1 in citrus results in early flowering with a reduction in the number and size of thorns (Pena et al., 2001). This ectopic expression suggests that upon flowering, vegetative determinacy is converted to floral determinacy. The observation made over 100 years ago of a ‘fruiting orange thorn’ also supports the idea that citrus thorns and flowers are alternate determinate states (Shamel and Pomeroy, 1918).

As with floral determinacy, vegetative determinacy can be plastic. In *Lycium ruthenicum*, a subset of tissue-cultured clones that were initially thornless developed thorns after transplantation, resulting in a mix of clones having determinate thorns while others having an indeterminate axillary bud at the same positions, regardless of being genetically identical siblings (Ke et al., 2025). Indeterminate axillary bud versus determinate thorn development can also be environmentally induced by drought in *Lycium ruthenicum*, and is associated with changes in DNA methylation (Yang et al., 2022).

## Conclusion

The control of meristem determinacy represents a fundamental developmental decision in plants, balancing the need for flexible organogenesis with the formation of terminal structures. Emerging comparative studies across diverse species reveal both conserved and divergent mechanisms controlling meristem fate, as well as variations in the temporal and spatial control of this fate. The balance in the temporal and spatial expression of determinacy factors such as FT and indeterminacy factors such as TFL1 can vary considerably in different species, which then modulates plant architecture (Moraes et al., 2019; Liu et al., 2021). In turn, the FT/TFL1 balance impinges on the regulation of conserved stem cell identity factors such as WUS. Understanding how the FT/TFL1 balance is regulated in distinct species, and how this balance impacts specific developmental outcomes, including the regulation of WUS, will be central in understanding the importance of flexibility and plasticity in shaping plant form.

Future studies should address several key questions: What molecular mechanisms enable reversible determinacy in plastic systems? How does reversible determinacy evolve in certain clades? How do long-range signals integrate local chromatin states to generate a stable meristematic fate? Although auxin-cytokinin gradients are critical for organogenesis in shoots, the combined influence of intrinsic physiological and extrinsic environmental signals on determinacy remains largely unexplored. To what extent are such signals instructive or permissive in determining stem cell identity and meristem determinacy? How do we leverage understanding of determinacy to improve crop architecture? As tools for single-cell spatial transcriptomics, live imaging, and genome editing continue to advance, we are well positioned to achieve a systems-level understanding of meristem fate control, bridging molecular mechanisms with tissue-level organization and whole-plant architecture. Hopefully, such insights will enable us to meet future agricultural challenges.
